# Individual Listener Preference for Strength of Single-Microphone Noise-Reduction; Trade-off Between Noise Tolerance and Signal Distortion Tolerance

**DOI:** 10.1177/23312165231192304

**Published:** 2023-08-01

**Authors:** Ilja Reinten, Inge de Ronde-Brons, Rolph Houben, Wouter Dreschler

**Affiliations:** 1 Clinical & Experimental Audiology, 26066 Amsterdam University Medical Centres location AMC, Amsterdam, the Netherlands; 2 Pento Audiological Centre, Utrecht, the Netherlands; 3 Pento Audiological Centre, Amersfoort, the Netherlands

**Keywords:** hearing loss, hearing aids, auditory perception, paired comparisons

## Abstract

There are large interindividual variations in preference for strength of noise-reduction (NR). It is hypothesized that each individual's tolerance for background noise on one hand and signal distortions on the other hand form this preference. We aim to visualize and analyze this so-called trade-off between noise attenuation and signal quality. Dutch sentences in stationary background noise were processed with different NR strengths. We used an NR algorithm that allows us to separate the positive effects of NR (noise attenuation) from the negative effects (signal distortion). Stimuli consisted of speech in noise with different degrees of (1) background noise, (2) signal distortions, or (3) both (i.e., realistic NR at different NR strengths). With pairwise comparisons, participants chose which stimulus they preferred for prolonged listening. Twelve listeners with mild to moderate hearing loss participated in the study. For all participants, a trade-off between noise attenuation and signal quality was found and visualized. The strength of preference was estimated with the Bradley–Terry–Luce choice model and was different for all individuals but in the same order of magnitude for distortion effects and noise effects. Strength of preference of realistic NR was smaller by a factor of ten. This study used a unique setup to capture the individual trade-off between noise attenuation and signal quality in NR. Disturbance from signal distortions is as important as disturbance from background noise for determining preference for NR strength. Individual listeners differ in their sensitivity to both factors and as a consequence in their preferred NR strength.

## Introduction

Single-microphone noise-reduction (NR) is an essential feature in most modern hearing aids. Noise-reduction algorithms attempt to improve the incoming speech signal in the presence of background noise. The algorithms do this by temporarily reducing hearing aid gain in the frequency bands that are dominated by background noise while preserving gain in frequency bands that are dominated by speech. It has been well established that NR can improve subjective listening experiences such as listening comfort and noise annoyance and is often preferred over no NR (Brons, Houben & Dreschler, 2014; Chong & Jenstad, 2018; Luts et al., 2010). For NR, there are currently no general fitting algorithms. Finding such fitting rules for NR is complicated because implementation details of NR algorithms appear to be different between manufacturers (Hoetink, Körössy & Dreschler, 2009) but are usually not known by the clinician or audiological researchers. Noise-reduction parameters are preset by the manufacturer, and in the fitting software, they can only be adjusted to a limited extent. Research has shown, however, that there is substantial variation across individuals in preferences for NR (Houben, Dijkstra & Dreschler, 2013; Neher, 2014; Reinten, de Ronde-Brons, Houben & Dreschler, 2019; Versfeld, Festen & Houtgast, 1999). Since poor performance in background noise is a commonly reported limitation of hearing aids (Bennett, Laplante-Lévesque, Meyer & Eikelboom, 2018), it might be possible to improve hearing aid user satisfaction if we can adequately adjust the NR to individual preferences. Thus, in order to improve user satisfaction we want to learn more about optimal individual fine-tuning of NR.

Given the complex nonlinear nature of NR with multiple interacting parameters, individual fine-tuning is challenging. Relevant parameters can roughly be categorized into dynamic parameters (e.g., several implemented time constants) and static parameters (e.g., number of frequency bands involved, amount of gain reduction). A commonly used static parameter for adjusting NR is the maximum amount of gain reduction, which we will refer to as NR strength. For NR strength, it has been previously shown that there are large interindividual differences in preference for NR strength (Brons, Dreschler & Houben, 2014; Houben et al., 2013; Neher & Wagener, 2016; Zakis, Hau & Blamey, 2009).

An explanation for the differences in preference for strength of NR between listeners lies in the inherent trade-off between noise attenuation and signal quality (Brons, Dreschler et al., 2014; Kubiak, Rennies, Ewert & Kollmeier, 2022; Reinten et al., 2019; Völker, Ernst & Kollmeier, 2018). Increasing NR strength has noise attenuation (less noise) as a positive effect, but signal distortion (Loizou & Kim, 2011) as a negative effect. One cause of signal distortion is that hearing aids have no a-priori knowledge of the mixture of incoming sounds, so an NR algorithm makes an imperfect estimation of the amount of noise and speech present in the signal. As a consequence, the reduction of noise inevitably affects speech. This leads to a lower speech quality but with less background noise. Another reason for distortion is that changing gain from time to time and in different frequency bands causes processing artifacts in the signal that might be audible. We will refer to this deterioration of the signal quality due to both imperfect estimation and processing artifacts as signal distortion. Note that we do not include the amount of background noise in the incoming sound mixture in the term signal distortion, although the noise can also be experienced as a form of distortion. It is believed that each individual compares two factors: how much noise they tolerate and how much NR signal distortion they tolerate. This trade-off between unwanted noise and unwanted distortion could steer individual differences in preferred NR strength.

**Figure 1.**
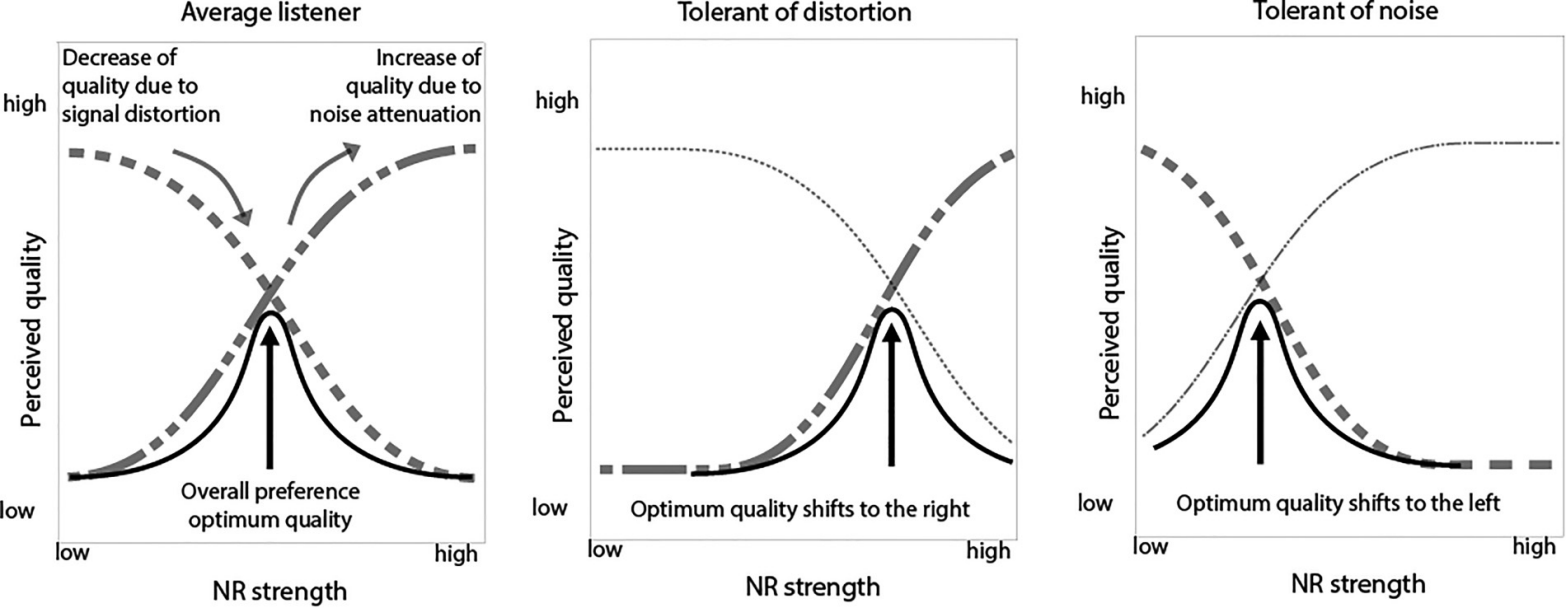
Hypothetical differences between individual preference for noise-reduction (NR) strength (continuous curves) in relation to the trade-off between the amount of noise (semi-dashed curves) and the amount of signal distortion (dashed curves). In the left figure, there is an equal balance between the two factors which makes that the optimum quality is in the middle. In the left and right figures, however, the optimum quality is shifted to the right and left, respectively, due to a different tolerance for either distortions or noise for the individual listener.

Differences in the individual weighting of the two factors are shown schematically in Figure 1. Each panel shows a possible effect of NR strength on the perceived quality for three criteria: noise attenuation (semidashed curves), signal distortion (dashed curves), and overall quality (continuous curves). An increase in NR strength causes an increase in perceived quality because the noise level reduces, but at the same time causes a decrease in perceived quality because of increased signal distortion. Plotted in this way, an optimal trade-off would be expected at the NR strength where both curves intersect. This is shown in the left panel of Figure 1. An individual listener, however, might be more tolerant for signal distortion than for noise level or vice versa. This can result in shifted curves for the signal quality, resulting in a changed value of the optimal NR strength as displayed in the middle and right panels of Figure 1. A listener who is tolerant of distortion prefers stronger NR in spite of signal artifacts. A listener who is tolerant of noise prefers less strong NR in spite of a higher noise level. The proposed relation between these individual traits and preference for NR settings has been introduced previously, for example, by Völker, Ernst and Kollmeier (2018) who used the terms “noise haters” and “distortion haters,” or Neher and Wagener (2016) who used the terms “NR haters” and “NR lovers.”

The possible influence of signal distortion on user preference has been investigated before. For example, Brons, Dreschler et al. (2014) designed a method to measure an individual's detection threshold for signal distortion caused by NR. They compared the distortion threshold to individual preferences for NR. No correlation was found between individually preferred NR strength and the individual's detection threshold for distortion. As expected, they did find that all participants preferred an NR strength above their distortion detection threshold, indicating that all participants tolerated some amount of audible signal distortion. They hypothesized that individual preference is based on a balance between wanted noise removal and unwanted audible signal distortion.

Similar results are found in a study by Neher et al. (2016). The authors categorized hearing aid users into one of two groups based on their preference for strong or weak NR and aimed to find predicting factors for belonging to either group. They did not find a significant predicting factor for belonging to either the strong or the weak NR group. However, in line with Brons et al. (2014), for all participants, the preferred NR strength was above the detection threshold for distortion.

In a recent study, Kubiak, Rennies, Ewert and Kollmeier (2022) tested the individual trade-off between a good signal-to-noise ratio and a distortion-free speech target for 30 participants with a wide range of hearing status (normally hearing to moderately impaired). Participants used a slider function to set target speech to a desired amplification level in quiet and in babble noise (two- or multitalker). Increasing amplification for speech meant not only a higher signal-to-noise ratio but also more speech distortion. Two types of signal distortions were used: peak clipping on the speech signal and dynamic range distortions. Using this method, the authors could classify their participants along a scale from the so-called “noise haters” to the “distortion haters.” Results showed that individual preferences were stable in time (high test–retest stability) as well as in distortion type (similar preference responses for the two induced signal distortion types). The distortions applied in this study differ from those introduced by an NR algorithm, but the findings of different listener types and stability in individual preference are also interesting in the light of preference for NR strength.

Currently, the extent to which audible NR distortion influences the HI listeners’ preference remains unknown. Additionally, the individual relation of noise tolerance and distortion tolerance has not been investigated directly for distortions induced by altering NR strength. In this study, we hypothesize that the optimal setting for NR strength is determined by an individual weighting of the tolerance for background noise and the tolerance for signal distortion. By using an artificial set-up, this study provides knowledge on the impact of noise attenuation and signal distortion that cannot be acquired with measurements from real hearing aids. Moreover, this setup is independent of inherent quality differences between different NR systems in real hearing aids (e.g., more or less distortions at the same level of residual noise). This study, therefore, contributes to our understanding of individual preferences for NR settings in HAs.

## Methods

We designed a listening test that uses paired comparisons to study the effects of NR strength on noise level and signal distortion separately. We used an NR system as described by Brons et al. (2014), which allows us to separate the positive effects of NR on noise level and the negative effects of NR distortion. Participants listened to three sets of speech in noise signals, where (1) only the noise level changes (stronger means less noise), (2) only the distortion level changes (stronger means more distortions), and (3) both effects are present due to applying realistic NR with different strengths (stronger means more noise attenuation and more signal distortions). The study was approved by the Medical Ethics Committee of the Amsterdam UMC, location AMC in March 2019, (NL68444.018.18).

### Participants

**Figure 2.**
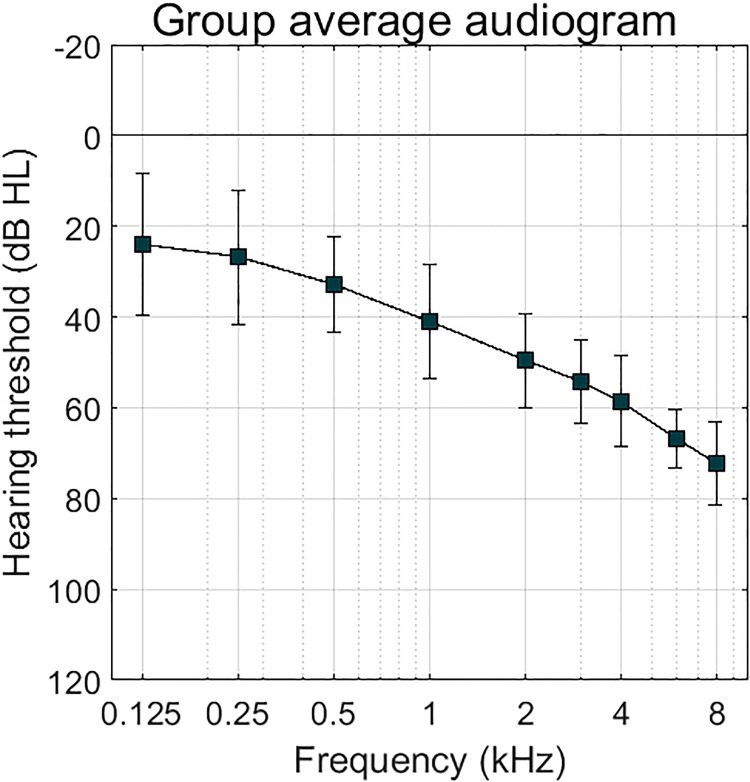
Averaged pure-tone thresholds of the tested ear of all 12 participants. Error bars show 95% confidence intervals.

Twelve participants with mild to moderate sloping sensorineural hearing loss were included. To reduce the possible effects of different pure-tone audiograms, we only included participants that had hearing thresholds classified between the N2 and N4 standard audiograms according to Bisgaard, Vlaming and Dahlquist (2010). In the listening tests, stimuli were presented monaurally, to the ear that was closest to the N3 standard audiogram. Averaged pure-tone thresholds with 95% confidence intervals of the tested ear are shown in Figure 2.

Participants were recruited from the database of the audiological center in the Amsterdam UMC, location AMC. All participants were native Dutch speakers. The participants had a mean age of 63.9 ± 12.7 years. There were four female and eight male participants. Ten participants were hearing aid users of whom seven had been using them for more than 5 years.

### Stimuli

The listening experiment took place in a soundproof booth and the stimuli were presented monaurally through Sennheiser HDA200 headphones. We used sentences with a female speaker from a set of recorded Dutch sentences (Versfeld, Daalder, Festen & Houtgast, 2000). Stationary noise with a matched speech spectrum (SSN) was added as background noise at a signal-to-noise ratio of +5 dB. An SNR of +5 dB was chosen because it resembles everyday situations (Smeds, Wolters & Rung, 2015) and it is in line with the work by Brons et al. (2014). At the start of the visit, the stimuli were calibrated such that the unprocessed speech-in-noise stimuli were presented at an average level of 65 dB(A). Subsequently, all stimuli were amplified according to the NAL-RP prescriptions for each individual listener (Dillon, 2001). This prescription rule provides linear amplification which restores speech intelligibility and improves the audibility of the stimuli that conform the individual hearing loss, comparable to what is offered in hearing aids. Given the dynamic range of our participants, no dynamic range compression was required. Moreover, linear amplification avoids interactions between NR and dynamic range compression (Brons, Houben & Dreschler, 2015), which can complicate the interpretation of the results.

### Noise-Reduction Algorithm

**Figure 3.**
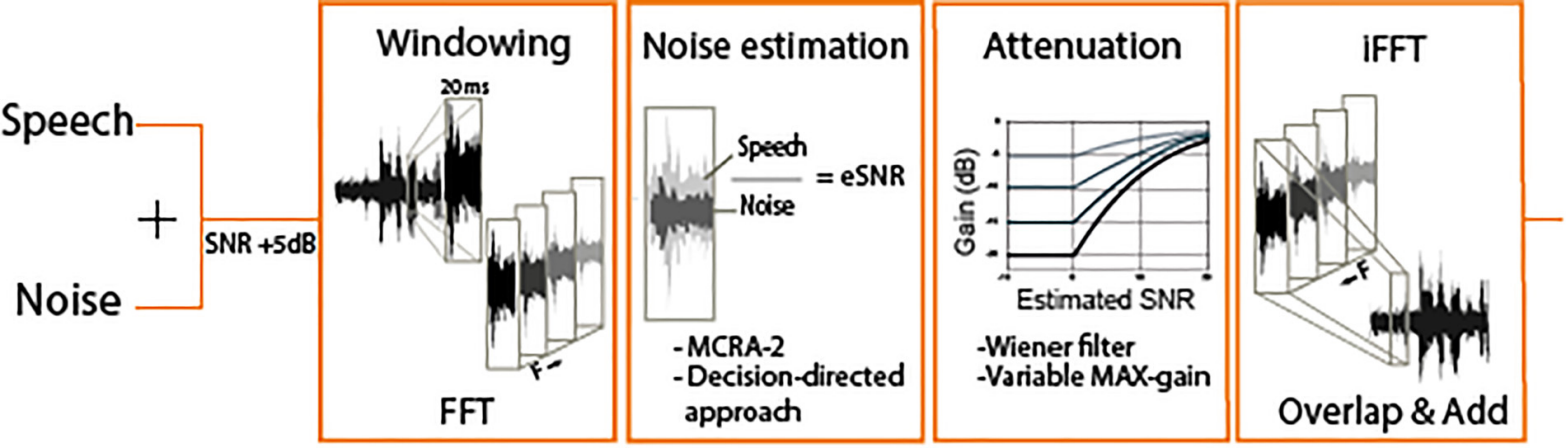
Simplified schematic overview of the noise-reduction (NR) algorithm that is used in the experiment. Each block shows an essential step in the algorithm. The NR strength is determined in the third block “Attenuation,” which allows a variable input of maximum gain (and thus maximum attenuation).

We processed all stimuli in Matlab (v R2018b). We used the NR from Brons et al. (2014), where a detailed explanation of the implemented algorithm can be found. In short, a Fast Fourier Transform (FFT) was performed on 20 ms segments formed by a Hamming window with 50% overlap. All stimuli were sampled at 44.1 kHz. An updated minimally controlled recursive algorithm (called MCRA-2 (Rangachari & Loizou, 2006)) estimated the amount of noise in the signal. The decision-directed approach (Ephraim, Malah & processing, 1984) was used for SNR estimation. This approach slows down the SNR estimate update so that the attenuation will not change radically from frame to frame. The noise-estimate was averaged over several frequency bins to resemble realistic hearing aid frequency channels. We used 15 frequency channels that were logarithmically divided between frequencies of 50 and 8000 Hz. Based on the estimated SNR (eSNR(f,t)), the attenuation (G(f,t)) was determined by a parametric Wiener filter:

(1) $$G(\;f,t)={\left(\frac{eSNR(\;f,t)}{\alpha +eSNR(\;f,t)}\right)}^{\beta }$$

with α = 1. The parameter β was used to vary the maximum attenuation strength. After gain reduction, an inverse FFT and the overlap and add method were used to recombine the overall signal. Figure 3 gives a schematic overview of the signal-processing scheme.

### Separating Noise Attenuation and Signal Distortion Effects

**Figure 4.**
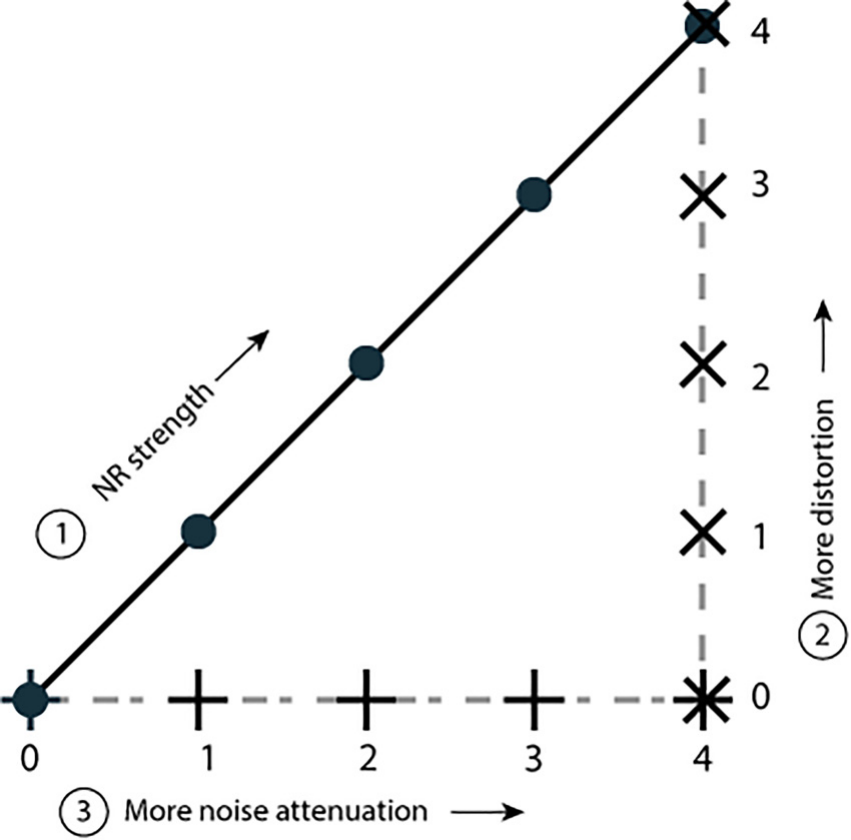
Graph representing all conditions used in this experiment. Realistic noise reduction with different strengths (0-1-2-3-4) is shown on the diagonal axis with round markers. The effect of noise reduction is split into a distortion-component on the vertical axis, and a noise-component on the horizontal axis. Conditions on the latter two axes do not resemble realistic noise reduction.

We separated the NR effects into an effect of reduced noise level and an effect of signal distortion. This separation with corresponding conditions is shown schematically in Figure 4. Realistic NR with different NR strengths on the diagonal axis can be split into two axes: a noise-axis (horizontal) and a distortion-axis (vertical). Symbols on the horizontal axis represent different degrees of noise attenuation, with a fixed distortion level (no distortion). Symbols on the vertical axes represent different degrees of distortion with a fixed noise level (low noise level).

**Figure 5.**
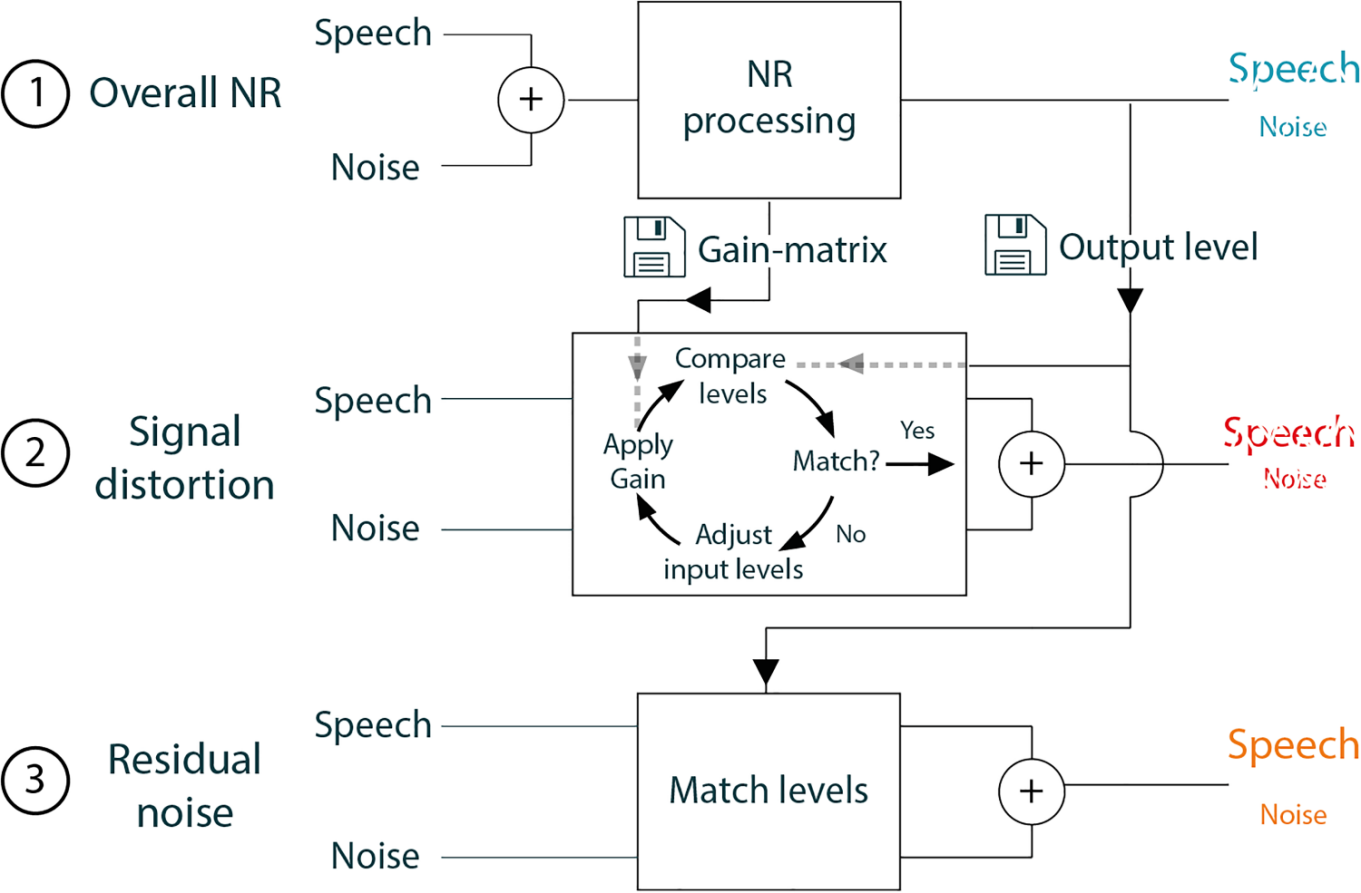
A simplified schematic overview that shows how the different stimuli were created.

We created stimuli that correspond to each of the markers shown in Figure 4. The creation of these stimuli occurred in three steps, schematically shown in Figure 5.

First, we created the conditions of the diagonal axis of Figure 4. Noise-reduction with different strengths (0-1-2-3-4) was applied to the input speech-in-noise signal. For each NR strength, processing was done on two consecutive lists of 13 sentences, where the first list served to stabilize the NR algorithm. This first list was removed after processing and not used in the listening tests.

After the processing, we had access not only to the processed speech in noise signals but also to the gain matrix that denotes the applied gain (or rather: applied attenuation with values between 0 and 1) for each time-frequency unit. We also recorded the output level of the speech and noise signals. We used the gain matrix to obtain the nonrealistic NR conditions (Figure 4 horizontal and vertical axes) by applying it to the input speech and noise signals separately.

An NR algorithm reduces the signal strength. To prevent unwanted differences in loudness within the distortion set in our listening experiment, we equated the level of the processed speech to the level of the processed speech at the strongest NR setting (i.e., 4). For example, when the distortion signal for NR strength 2 was created, the input speech and noise sound levels were reduced in such a way that after multiplying the signals with the gain-matrix of NR strength 2, the output sound levels of noise and speech were equal to the sound level of NR strength 4. The amount of distortion, however, was appropriate for NR strength 2.

To obtain stimuli with different amount of noise, we matched the SNR of the input signal to the output SNR of the NR processing, without other effects of that NR processing. These stimuli are represented by the symbols on the horizontal axis in Figure 4.

Noise-reduction reduces the sound level of the speech-in-noise signal. That means that for stronger degrees of NR strength not only the sound level of the noise but also the sound level of the speech signal decreases. To avoid the unwanted influence of loudness in the paired comparison experiment, we amplified all stimuli to the RMS of the original speech and noise mixture (NR strength 0, no signal processing). Note: this level correction is different from the correction applied in step 2 to create the distortion signals. Because signals with an equal RMS might still have a different loudness, we additionally applied level roving, as described below.

We used values of NR strength between 6 and 24 dB, with 6 dB steps. These values were based on previous research with this NR algorithm (Brons, Dreschler et al., 2014). We chose values around the mean preferred NR strength for HI listeners (11.9 dB ± 5.3 dB), with steps that roughly correspond to their mean distortion detection threshold (6.7 dB ± 3.3 dB).

Additionally, the values for NR strength that we chose also correspond with realistic values of NR strength in hearing aids (0–24 dB) (Chung, 2004). Table 1 gives an overview of the stimuli. Note that for the distortion axis, a higher number means more distortion, and for the noise axis a higher number means less noise.

**Table 1.** Labels for All Conditions in the Experiment, with Corresponding Maximum Attenuation.

A-max	Realistic NR	Distortion axis	Noise axis
0 dB	NR0 (=N0)	D0 (=N4)	N0 (=NR0)
6 dB	NR1	D1	N1
12 dB	NR2	D2	N2
18 dB	NR3	D3	N3
24 dB	NR4 (=D4)	D4 (=NR4)	N4 (=D0)

**Figure 6.**
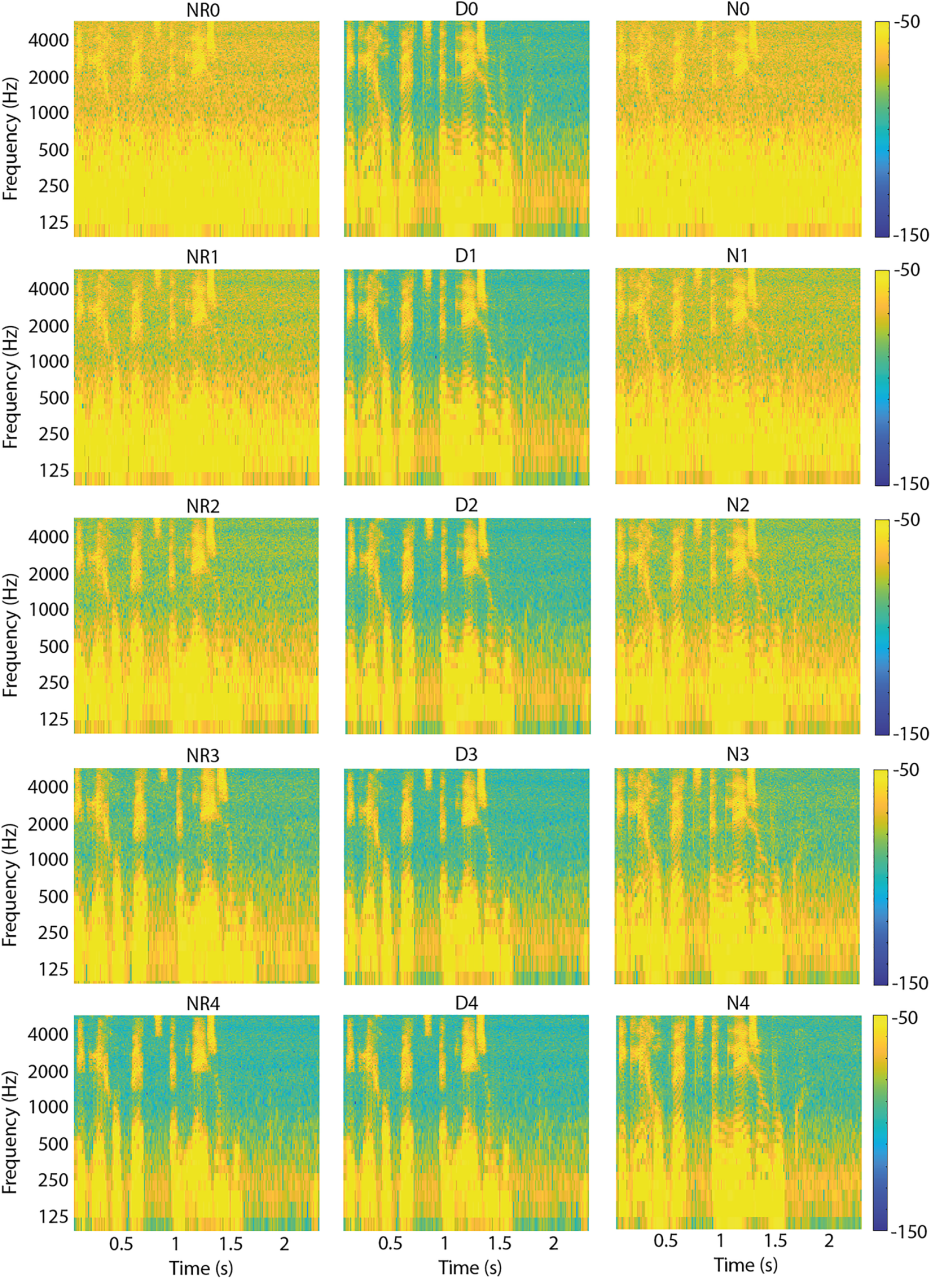
Spectrograms of all conditions used in this experiment. Each spectrogram shows time (in seconds) on the horizontal axis and frequency (in Hertz) on the vertical axis. The power level for each time-frequency unit is shown with different colors. Yellow shows the highest power level (−50 dB) and blue the lowest (−150 dB).

**Figure 7.**
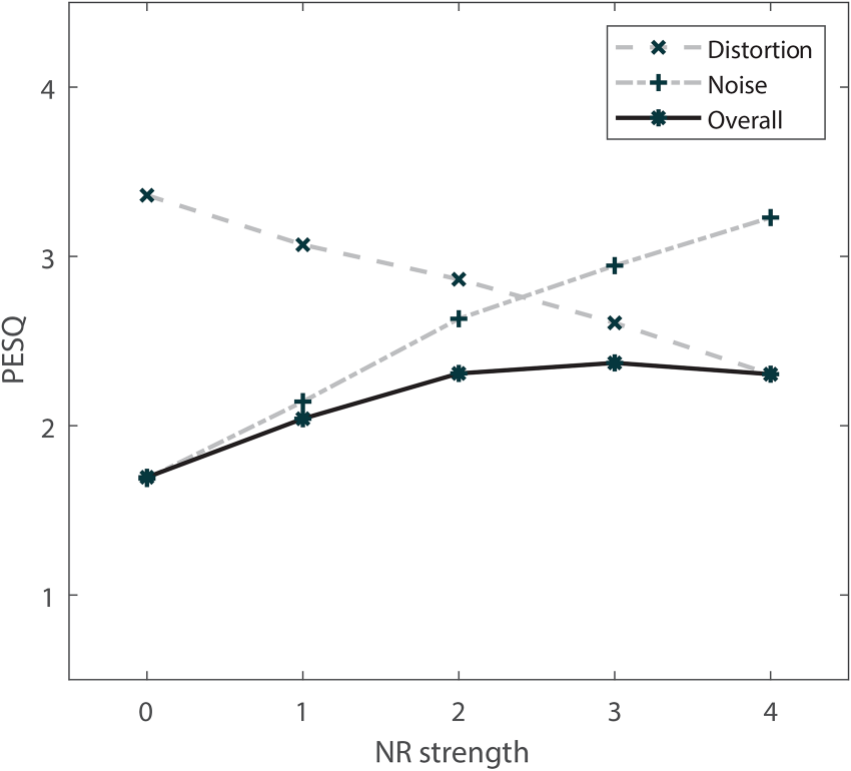
Perceptual Evaluation of Speech Quality (PESQ) scores for each condition of the experiment. The vertical axis shows the PESQ scores. The horizontal axis shows noise-reduction (NR) strength of the overall stimuli and corresponds to the different degrees of signal distortion and noise attenuation of the distortion and noise stimuli.

Figure 6 shows time-frequency spectrograms of the conditions as specified in Table 1. For these spectrograms, one sentence was used as an example. The horizontal axis shows time in seconds, and the vertical axis shows frequency in Hertz. The different shades of gray in the spectrogram denote the power level per time-frequency unit. Note that, as is shown in Table 1, some conditions overlap (e.g., NR0 is equal to N0). To get an objective measure of signal quality, we calculated the Perceptual Evaluation of Speech Quality (PESQ) scores for all conditions. Perceptual Evaluation of Speech Quality scores have a possible range from 1 (bad) to 4.5 (no distortions) (Rix, Beerends, Hollier & Hekstra, 2001). We used a set of 13 concatenated processed sentences. Calculation was done with the matlab script “pesq” provided by Loizou (2013). Figure 7 shows the PESQ scores of all conditions.

### Paired-Comparison Listening Test

A complete design for paired comparisons was used: all 12 conditions were compared to all others. This resulted in a total of 66 unique stimulus pairs. Three runs of comparisons were done, resulting in a total of 198 comparisons per listener. We based our choice of three runs on the results of Houben et al. (2013). For a similar NR algorithm, they concluded in their paired comparison experiment that three runs were the optimal amount of repetitions for estimating the preferred NR strength. The order of comparisons was balanced using a Latin square design. The order of presentation of the stimuli within each comparison was alternated, resulting in two AB and one BA comparisons for half of the participants and one AB and two BA comparisons for the other half.

Listener preference was determined with a two-interval, two-alternative forced choice paradigm. The participants were asked to listen to a sentence processed in two different ways and to make a choice between the two processing types based on the following question: “Imagine that you will have to listen to these signals all day. Which sound would you prefer for prolonged listening?.” We applied a balanced random level roving of 1 dB, 0 dB, or −1 dB to one of the stimuli of each comparison in order to reduce possible loudness effects.

### Data Analysis

We combined all three condition sets in a single full-comparison design to allow preference ranking over all conditions, irrespective if the sentences belong to the set with realistic NR, only noise attenuation, or only distortion inductions. The paired comparison data were expressed as win counts by counting how often a condition was chosen over all other conditions. This procedure resulted in a single ranking of all conditions. We represented this in a figure with the level of NR strength (or the equivalent strength in the noise and distortion axes) on the horizontal axis and the win counts on the vertical axis. Each condition set is represented by a different curve, similar to the hypothetical examples in Figure 1.

The strength of preference was modeled with the commonly used Bradley–Terry–Luce (BTL) choice model (Zimmer & Ellermeier, 2003). All modeling was done in Matlab with a script written by Wickelmaier and Schmid (2004). We used the model to quantify the difference in preference between our conditions. The model does this by converting win counts (preference ranking) into preference values (worth) on a ratio scale. A worth that is twice as high indicates that the condition is two times as preferred as the other condition.

## Results

**Figure 8.**
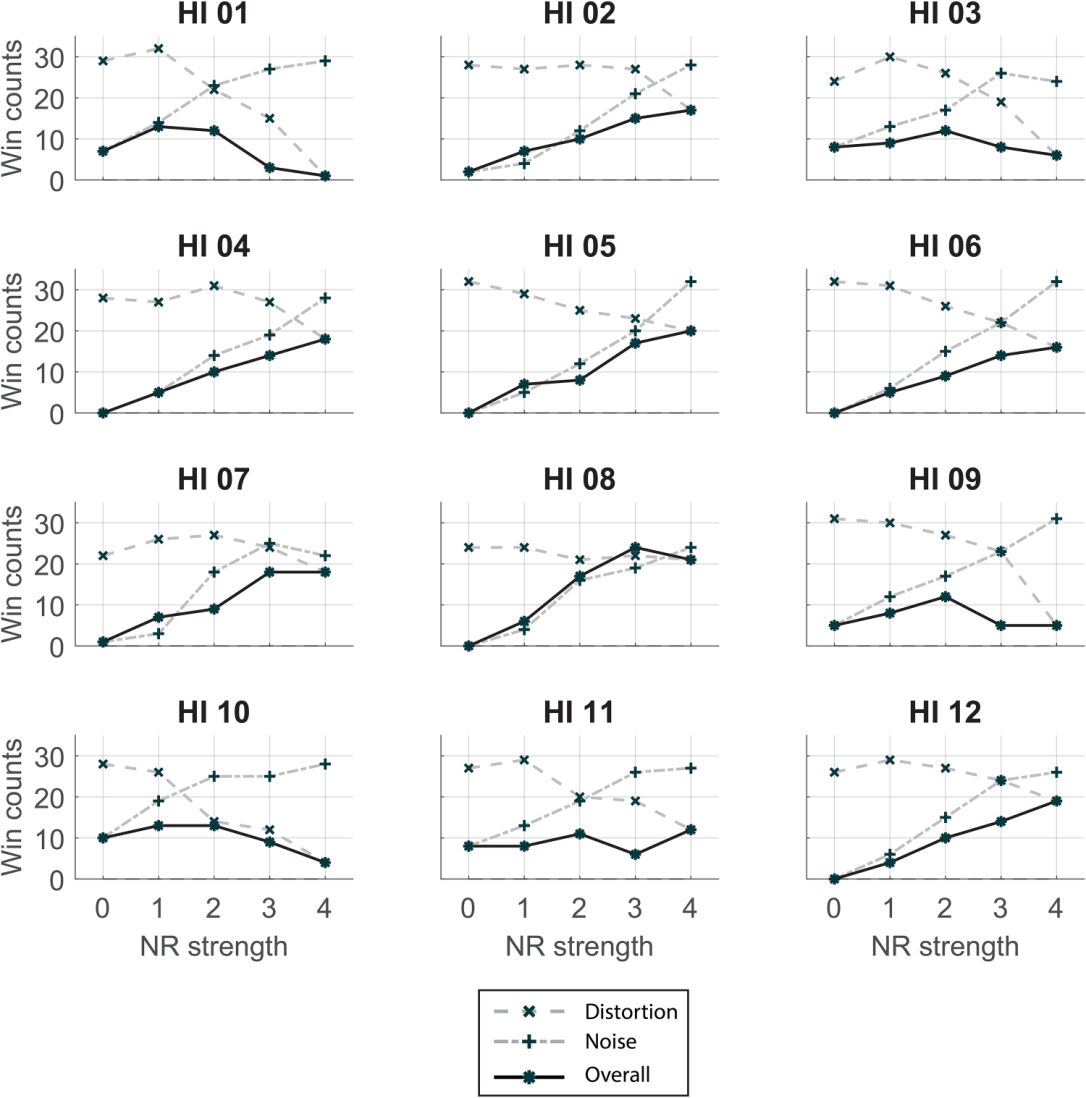
Trade-off plots for each individual HI listener. Per subplot, the win counts in the paired comparisons are shown for each sound category (only distortions, only noise, both), see the legend for identification of the curves. The horizontal axis shows noise-reduction (NR) strength of the overall curve and corresponds to the different degrees of signal distortion and noise attenuation.

For each HI listener, a trade-off plot was made, shown in Figure 8. Each subplot shows the win counts on the vertical axis. The horizontal axis shows the NR strength for the realistic NR setting and corresponds to the different degrees of distortion and noise as shown in Figure 4 and Table 1. The different processing sets (only distortion, only noise attenuation, or both) are shown by different curves. A higher win count means that this condition was chosen more often over conditions with a lower win count. For instance, we see for all participants that the win counts for the noise conditions increase with increasing NR strength. This relation indicates that listeners prefer conditions with less background noise. Similarly, win counts (and thus preference) for the distortion conditions decrease with higher NR strength. However, the rate of change, the gradient of decreasing win counts, differs between participants. The shape of the win count line for the realistic NR strength (“overall” curve) also differs between participants, However, for all participants the overall curve is lower (less preferred) than the other two curves meaning that the conditions with only noise or only distortion effects were chosen more often than the conditions with both effects (realistic NR).

To see how consistent participants were in their preference, we calculated the coefficient of consistency based on the number of circular triads (inconsistent combinations of preferences, for instance when A is preferred over B, B is preferred over C, but C is preferred over A). We calculated the coefficient of consistency for each participant and each repeat of the paired comparisons (Kendall & Smith, 1940). Additionally, for each participant, Kendall's coefficient of agreement (W) was calculated between the different repeats of the paired comparisons. The first three columns of Table 2 give the coefficients of consistency, ζ, specified per participant and for each set of 66 paired comparisons (since all comparisons were repeated two times). A higher value (between 0 and 1) indicates a lower number of inconsistent combinations. The fourth and fifth column of Table 2 gives Kendall's coefficients of agreement, W, and corresponding *p*-values for each participant. For interpreting W, a value closer to 1 means a higher agreement between the different runs.

**Table 2.** Coefficients of Consistence, ζ, of Each Repeat of Paired Comparisons, and Kendall's Coefficients of Agreement, W, with *p*-values for Each Participant.

Participant	ζ 1^st^ repeat	ζ 2^nd^ repeat	ζ 3^rd^ repeat	W	*p*
HI01	0.89	0.96	0.89	0.95	<.01
HI02	0.84	0.84	0.84	0.93	<.01
HI03	0.56	0.78	0.8	0.79	<.01
HI04	0.94	0.97	0.93	0.93	<.01
HI05	0.84	0.86	1	0.95	<.01
HI06	0.91	0.96	1	0.94	<.01
HI07	0.79	0.77	0.81	0.89	<.01
HI08	0.72	0.74	0.8	0.63	.04
HI09	0.89	0.84	0.9	0.95	<.01
HI10	0.56	0.66	0.61	0.81	<.01
HI11	0.81	0.49	0.71	0.78	<.01
HI12	0.83	0.87	0.96	0.90	<.01

Using the BTL choice model, worth parameters were estimated for each condition. Table 3 shows the goodness-of-fit for each model, per HI listener. The *p*-value represents the probability that the model outcomes correctly describe the underlying data. χ^2^ indicates how much the observed values differ from the predicted values. The models can be accepted if the value for χ^2^ is lower than 68.8, that is, the critical χ^2^ value for our degrees of freedom and chosen alpha (0.1). This alpha value is chosen because it is proposed by Wickelmaier and Schmid (2004). All BTL models fit the data well and all models were accepted. However, the *p*-values of HI06, HI09, and HI10 indicate a low probability that they correctly describe the underlying data which are why for these participants the results of Figure 8 (absolute win counts) should be leading for interpreting their answers.

**Table 3.** Goodness-of-Fit of the Bradley–Terry–Luce Model for All Individual Listeners.

Participant	HI01	HI02	HI03	HI04	HI05	HI06	HI07	HI08	HI09	HI10	HI11	HI12
*p*	>.99	>.99	.7	>.99	.97	.27	.94	>.99	.4	.34	>.99	.99
χ^2^(55)	17.6	28.7	49.1	30.8	36.9	60.7	39.8	22.6	56.8	58.7	31	34.7

**Figure 9.**
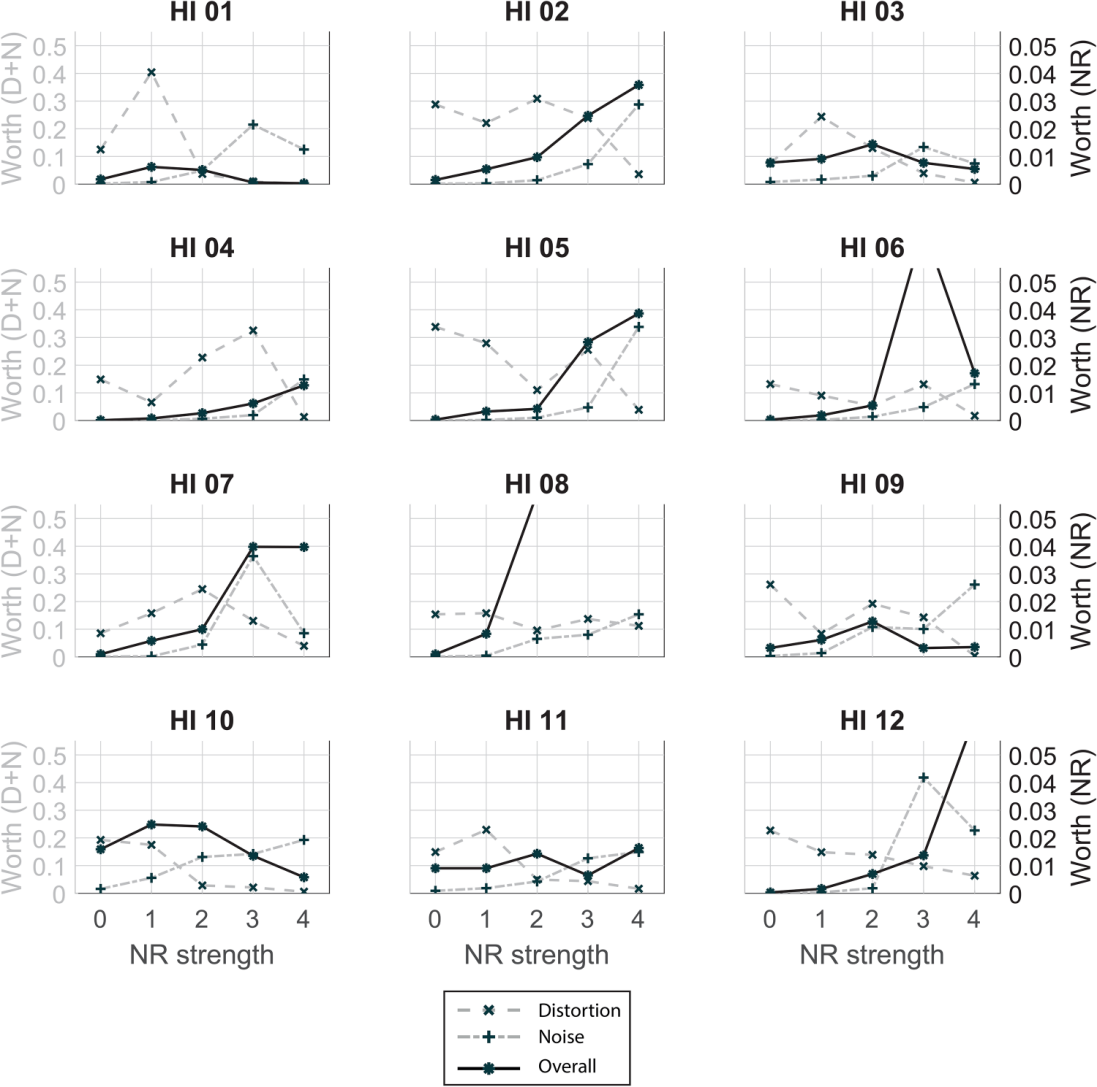
Estimated worth parameters for each individual HI listener. Per subplot, the estimated worth parameters from the Bradley–Terry–Luce (BTL) model are shown for each sound category (only distortions, only noise, both), see the legend for identification of the curves. The horizontal axis shows noise-reduction (NR) strength of the overall curve and corresponds to the different degrees of signal distortion and noise attenuation. Note that the vertical axis has two scales shown on the left and right side, respectively. The left side shows the scale for the worth parameters of the distortion and noise processing sets. The right side shows the scale for the worth parameters of the realistic NR conditions.

**Figure 10.**
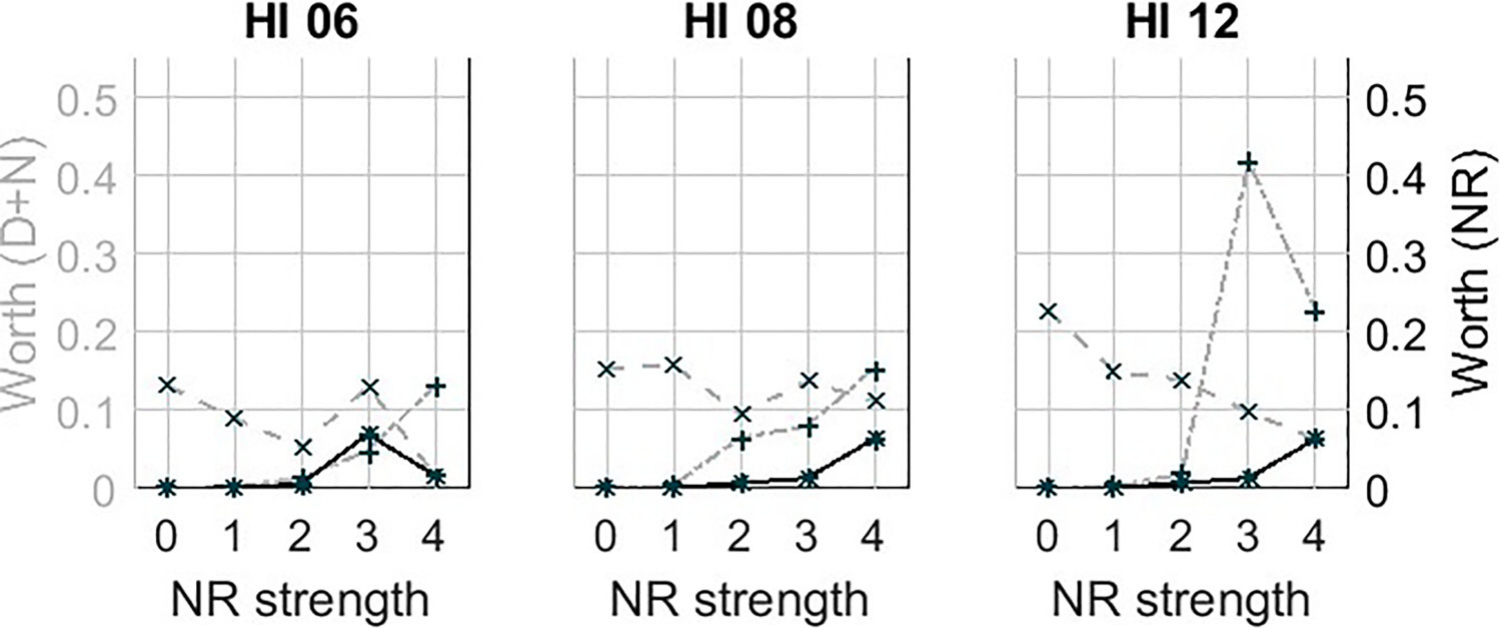
Estimated worth parameters for HI06, HI06, and HI12, similar to the plots in Figure 9 but with a different scale for the vertical axis.

Figure 9 shows trade-off plots, this time with the estimated worth parameters, from the BTL model for each participant. Note that the vertical axis has two scales shown on the left and right side, respectively. The left side shows the scale for the worth parameters of the distortion and noise processing sets. The right side shows the scale for the worth parameters of the realistic NR conditions. The values on the vertical scales give information on the strength of preference of the different conditions. Figure 10 shows the same trade-off plots of HI06, HI08, and HI12, with a different scale since not all results are visible in Figure 9.

## Discussion

The current study explored the individual trade-off between noise attenuation and signal quality that is assumed to underlie the preference for NR strength. We hypothesized that individuals differ in susceptibility to either noise or distortion. Our artificial NR algorithm allowed us to split noise and distortion effects. This is clearly shown in the trade-off plots visualized in Figure 8. For most HI listeners, the trade-off is obvious: a decrease in win counts on the distortion axis is accompanied by an increase in win counts on the noise axis. The trade-off plots with the estimated worth parameters in Figure 9 confirm these findings. The worth parameters give information on the value of preference for each condition. For nearly all participants, the overall curves have an optimum around the intersection of the distortion and noise curves. This suggests that the overall preference for NR strength is indeed individually balanced between distortion tolerance and noise tolerance.

In the trade-off plots in Figures 8 and 9, we can visually distinguish between different types of listeners. The most striking observation is that the overall preference curve of NR strength increases until the maximum NR strength for seven of the participants (HI02, HI04, HI05, HI06, HI07, HI08, and HI12), that is, the stronger the better. For these participants, the overall preference closely follows the trend of the results in the noise curve. This suggests that for them, noise attenuation is most important in determining preference for NR strength. For the other participants, their preference is more balanced between distortion and noise effects which suggest a lower tolerance for distortion effects. These observations are visible in both presentations of the results (Figures 8 and 9). The noise curves for all participants are fairly similar which implies that everybody can hear and appreciate the effect of noise attenuation. The distortion curves show more variability between participants. For some listeners, this curve slopes down quickly (HI01, HI10, and HI11) while for others the curve is rather flat (HI02, HI04, HI07, and HI08). This implies that some listeners hear and appreciate a better sound quality while others either hear the signal distortions but are not disturbed by them or they do not hear them at all. In general, the effect of noise attenuation is more clear than the effect of signal distortions. This is in line with the results of Brons, Houben and Dreschler (2014) who found a greater effect for noise annoyance than for speech naturalness in their paired comparison listening test evaluating different NR algorithms.

Previous studies have tried to relate preference for NR strength or other NR parameters to personal factors such as hearing status, tolerance for background noise, or executive functions, but obvious explanatory factors were not found (Arehart, Souza, Kates, Lunner & Pedersen, 2015; Brons, Dreschler et al., 2014; Houben et al., 2013; Neher & Wagener, 2016). In this study, we did not aim to find underlying individual traits that cause the difference in preference and the number of participants does not allow us to perform statistical analysis on personal factors. Therefore, we should be reluctant to use the current results for strong statements or conclusions on common grounds between different listener types. We did, however, notice that all participants who were tolerant of distortions (HI02, HI04, HI05, HI07, HI08, and HI12) were using their hearing aids for over five years, while the other participants with the exception of HI11 were using their hearing aids for a shorter amount of time or not at all. This suggests that adaptation to hearing aid signal processing possibly makes a listener more tolerant of signal distortions induced, which is an idea that to our knowledge has not been investigated before. As the current study does not allow us to draw firm conclusions on this, we propose to take it in future studies.

Since all conditions were tested in one paired comparison test and analyzed with the same model, the strength of preference of all components can be compared with each other. It is notable that the worth parameters of the conditions with only distortion or noise are about a factor 10 higher than those of the condition with both distortion and noise (Figure 9), for most listeners. This indicates that the combined effect of both noise and signal distortions lowers the preference substantially. In all conditions determining the signal distortion axis, the noise level is low (at the level of maximum NR strength), and in all noise conditions, there is no speech distortion. The difference in magnitude of worth parameter shows that listeners clearly appreciate this advantage in one aspect. Moreover, the results show that the decrease in preference due to distortion of the signal is in the same order of magnitude as the decrease in preference due to the presence of background noise. Thus, for individual fitting of NR strength, the tolerance for distortions should be taken into account as well as the tolerance for background noise.

In a study by Brons et al. (2013), participants judged four NR algorithms from commercial hearing aids based on three sound properties: speech naturalness, noise annoyance, and overall preference. The authors then applied a regression analysis in order to find the predictive power of speech naturalness and noise annoyance on overall preference. In their analysis, it was observed that individuals weigh the factors speech naturalness and noise annoyance differently, in forming overall preference. Our results confirm this finding. Although all individual results show a trade-off between noise attenuation and signal quality, the weighting of both factors differs for each individual resulting in a different optimal NR strength. An important difference between their and our experiment is that participants in our experiment were not asked to judge one sound sample on different aspects, but were asked to directly compare sound samples representing the different aspects. That means that for the interpretation of the results we do not have to take into account the subjective notion of “speech naturalness” which might very well be different amongst HI listeners (Huber et al., 2018; Marzinzik, 2001). Therefore, we expect that in the current study differences between individuals in their distortion curve should be more directly linked to their distortion tolerance.

When drawing conclusions from the results, it is important to know how consistent and stable participants are in their preference. Table 2 shows the coefficients of consistency as well as Kendall's coefficients of agreement for each participant (Kendall & Smith, 1940). The coefficient of consistency is 1 if there are no inconsistencies in the responses and 0 when the number of inconsistencies is maximal. Since each participant had three rounds of paired comparisons in their listening test, we calculated coefficients of consistency for each repeat. There is no clear cutoff value for ζ below which the participant is too inconsistent, as different studies use different values (Amman & Greenberg, 1999; Weber, 1999). However, the values in Table 2 represent high levels of consistency, except for HI10 which might be considered inconsistent according to some interpreters.

Kendall's coefficients of agreement (W) were determined for each participant between the different repeats of the paired comparison experiment, in order to explore how stable participants were in their preference. All *p*-values for W were smaller than the significance level .05, which indicates that there is an intrarater agreement. All participants had a good concordance between measurements, with the exception of HI08 who had a moderate concordance. Thus, between the repetitions of paired comparisons in the listening test, our participants were stable in their preferences. This, however, does not imply that preferences are also stable when longer amounts of time have passed. But, several studies have already shown that preference for NR strength is stable in time, (Kubiak et al., 2022; Neher & Wagener, 2016) from which we assume that this holds true also for the preference responses in the current experiment. Individual preference for NR strength remains difficult to predict. A paired comparison experiment such as that used in this study is informative, although lengthy, and therefore, not useful in clinical practice. A well-known alternative method that is related to individual preference is the acceptable noise level (ANL). The ANL was first introduced by Nabelek, Tucker & Letowski (1991) as a measure to find the lowest SNR a listener would tolerate for prolonged listening. In a short test, the listener indicates her/his most comfortable level of running speech and thereafter the highest tolerable level of background noise. The ANL is defined as the difference between these two levels. It has been shown that NR can positively influence an individual's ANL (Fredelake, Holube, Schlueter & Hansen, 2012). Unfortunately, the measure has not been proven to be a suitable predictor for preferences for NR settings thus far (Neher & Wagener, 2016; Recker, Goyette & Galster, 2020). Given that the current results reinforce the idea that noise is not the only factor that determines the preference for NR strength, a shortcoming of the ANL might be that it does not account for signal distortion tolerance. It might be useful to develop a comparable measure that also incorporates an acceptable amount of distortion. The method used by Kubiak et al. (2022) who use a slider across a range of distortions might be a starting point for developing such a measure. The processing techniques used in the current experiment could be a useful addition to this.

The current study does have its limitations and the conclusions apply to the conditions tested only. Firstly, we used an artificial NR algorithm. It is difficult to judge whether the type of distortions used in our experiment are representative of hearing aid processing since details of algorithms in hearing aids are usually not shared. However, with the choices we made in the algorithm, we tried to mimic realistic NR processing (i.e., range of NR strengths, number of channels, using a Wiener filter). Signal distortions from other implementations could be perceived differently. However, we believe that our main results are generalizable. Specifically, we feel that the showed importance of both signal distortions and background noise as factors that determine user preference, are important for real hearing aids. A trade-off between noise attenuation and signal quality is inevitably present in all current NR algorithms.

Secondly, we only used stationary noise. Although this type of noise is a good starting point for investigating the trade-off between noise attenuation and signal quality, it is not representative of other common and more complex types of background noises such as speech babble. Given the complexity of other background noises such as speech babble, we can assume that NR algorithms have more difficulty separating speech from noise which can result in more signal distortion effects. We suggest the use of different kinds of background noises in future studies to further investigate individual preferences in relation to the trade-off between noise attenuation and signal quality. Finally, it should be noted that the several axes in the trade-off plots have overlapping data points by definition. Therefore, regardless of the results, the upper edges of the trade-off plots (D0 = N4) and the lower edges of the trade-off plots (NR0 = N0, NR4 = D4) aid in the visualization of the trade-off which we are looking for.

## Conclusions

The current study visualized individual preferences for NR strengths in relation to the trade-off between unwanted signal distortion and unwanted background noise. We used a unique experimental design, in which these two components of preference could be compared directly. This method allowed us to show that disturbance from signal distortions is as important as disturbance from background noise for determining preference for NR strength. Individual listeners appear to differ in their sensitivity to signal distortions and amount of background noise. If one attempts to design a fitting rule for optimizing NR for an individual, it may be worthwhile using a measure that incorporates both signal distortion tolerance and background noise tolerance.
